# Anorexia Nervosa and Refeeding Syndrome. A Case Report

**DOI:** 10.1100/tsw.2007.78

**Published:** 2007-03-09

**Authors:** Kohji Azumagawa, Yukiko Kambara, Naohisa Kawamura, Yoshito Takenaka, Takeshi Yamasaki, Hidetaka Tanaka, Hiroshi Tamai

**Affiliations:** ^1^ Department of Pediatrics, Yao Tokushukai General Hospital, Osaka, Japan; ^2^ Department of Pediatrics, Osaka Rosai Hospital, Osaka, Japan; ^3^ Department of Pediatrics, Osaka Medical College, Osaka, Japan

**Keywords:** anorexia nervosa, refeeding syndrome, disseminated intravascular coagulation, heart failure, liver failure

## Abstract

This is a case story of a 14-year-old girl with severe anorexia nervosa (AN) (158 cm, 28 kg, –44.1% ideal body mass index), admitted with purpura, edema, and general fatigue. We treated her carefully and paid particular attention to prevent development of refeeding syndrome (RS), and her body weight increased satisfactorily. However, RS (edema, hypoalbuminemia, and heart failure) occurred despite careful treatment. We used albumin and diuretics for treatment of RS, but severe liver damage resulted. RS was aggravated by the medical treatment. More attention should have been paid to her weight gain and medical treatment should have been initiated more slowly to prevent dramatic changes in the patient's fluid and electrolyte status.

## INTRODUCTION

Anorexia nervosa (AN) is a syndrome involving food restriction and extreme thinness due to psychological factors. It occurs particularly in adolescent girls and can cause significant medical complications in every organ system in the growing and developing body. It is a disease entity characterized by emaciation due to a patient's irrational pursuit of thinness. Prolonged food restriction causes severe malnutrition and various complications. The primary features of this syndrome are significant voluntary weight loss, hypometabolism, amenorrhea, distorted body image, and excessive exercise despite cachexia. Moreover, more severe AN in adolescence causes cardiovascular complications, alterations in linear growth, impaired bone mineral accretion, structural and functional brain changes, and refeeding syndrome (RS). However, reports about disseminated intravascular coagulation (DIC) complicated with AN and severe liver failure associated with RS are few.

When a malnutritioned patient with various clinical complications begins nutritional support, care must be taken to avoid RS. RS is defined as the occurrence of severe fluid and electrolyte shifts and dysfunction of various organ systems, for example, cardiovascular, neurologic, and hematologic complications. In RS, there is a rapid reversal in the insulin, thyroid, and adrenergic endocrine systems, shifting toward anabolism, resulting in significant morbidity and mortality. In the early stage of electrolyte deficiency, the body's hydration and nutrition status are masked by renal deficiency and acidosis, causing the status to appear normal outwardly, so it is sometimes difficult to prevent falling into RS[1].

In this article, we report a case of severe AN with coagulation disorder that developed RS in spite of careful monitoring and aggravated by medical treatments.

## CASE REPORT

A 14-year-old girl was admitted to our hospital with purpura, edema, and general fatigue. Her early medical history was not significant, but in the sixth grade of elementary school, she was teased by her peers who said she was fat (155 cm, 57 kg, +19% of her ideal body mass index[BMI]) and this led her to pursue dietary restriction. After about 6 months of dieting, her body weight had decreased from 57 to 52 kg, and amenorrhea occurred. Nine months later, she had lost 7 kg (to 45 kg) and 1 year later, she weighed 39 kg, at which time she was referred to a neuropsychiatric unit. There she was diagnosed as having AN of the restricting type (AN-R) according to the criteria DSM-IV[2], after organic causes of the weight loss were excluded. Although she was forced to take meals for 3 months to restore her body weight, she was hardly able to take any food and her body weight decreased further to 28 kg (–44.1% of her ideal BMI). After an episode of bronchitis, ecchymoses as well as petechiae appeared over her entire body, and edema of the legs occurred. At this point, the patient was admitted to our hospital.

Physical examination on admission revealed marked emaciation with a body weight of 28 kg. She was 158 cm in height and her fat ratio was below the sensitivity level. Her body temperature was normal, but she had bradycardia (54 beats/min), ecchymoses and petechiae over her entire body, and edema in her lower legs. Her secondary sex characteristics: breast was sexual maturity rating SMR 3, pubic hair was SMR 3, and menophania began at the age of 12 years old. Her total protein (5.2g/dl), prealbumin (10.5mg/dl), transferrin (82mg/dl), retinol binding protein (2.1mg/dl), phosphate (1.5mg/dl) and total cholesterol levels were low; her blood urea nitrogen (58.4mg/l) level was high. Liver dysfunctions (AST 329IU/l, ALT 321IU/l) were noted as well as coagulation disorders (PT26.9%, APTT 28.8sec, HPT 31.3%). QTc prolongation was apparent on her electrocardiogram, her ejection fraction was 62%, and pericardial effusion was detected on her echocardiogram. No electroencephalographic abnormalities were found, but brain atrophy was seen on the head CT scan, and results of the Baum test showed subtle psychological regression. On the basis of the clinical signs and laboratory data described above, we diagnosed her as severe AN combined with early DIC. So parenteral nutrition was started carefully so as not to cause her to fall into RS by peripheral injection, which was 120 kcal/day solution containing sufficient phosphate, amino acids, and vitamins in addition to 600 kcal/day of enteral nutrition via nasopharyngeal tube. Despite these nutritional interventions to prevent development of RS, the patient's platelet count decreased, so nafamostat mesilate was prescribed. These treatments were effective for her general condition, edema, and petechiae; her body weight began to increase 9 days after admission. Her energy intake was gradually increased to 1,000 kcal/day orally and to 300 kcal/day (750 ml/day) by peripheral injection. Although her weight increased to 32 kg by hospital day 18, significant edema in her legs reappeared, hypoalbuminemia occurred (2.9 g/dl), and echocardiography revealed pericardial effusion and dilatation of the inferior vena cava, which indicated development of RS. Administration of 2.5 g albumin and furosemide were prescribed to improve these symptoms, but severe liver damage (AST 1914 IU/l, ALT 1582 IU/l) ensued, that is, RS was aggravated by this treatment. She was transferred to the intensive care unit of Osaka Rosai Hospital, where intravenous hyperalimentation was performed and dopamine hydrochloride was administered for 3 days, resulting in improved circulatory conditions. Upon transfer, serum phosphate concentration was 1.5 mg/dl. Intravenous hyperalimentation with phosphate was performed carefully while fluid and glucose tolerance was monitored. Consequently, her serum phosphate level increased to 2.1 mg/dl 2 days after transfer and to 3.2 mg/dl 4 days after transfer, stabilizing at 3.2–3.7 mg/dl for the remainder of the recovery period. Three days after she left the intensive care unit, hyperalimentation at 800 kcal/day was provided and after 7 days, she was able to take 1,000 kcal/day orally. Liver function improved consistently with improvement in the serum electrolyte shift and normalized within 3 weeks. Three months after admission to Osaka Rosai Hospital, the patient's body weight had increased to 35 kg, and there was no clinical symptom of RS.

## DISCUSSION

Our case was a severe AN complicated with bleeding tendency. She also showed some features of AN, such as significant weight loss (28 kg, –44.1% of ideal BMI), bradycardia (54 beats/min), and edema of the legs. The ecchymoses and petechiae over her entire body revealed a bleeding tendency caused by AN. In AN, the platelet count generally reduces due to bone marrow hypoplasia and/or hypophosphatemia. In our case, neither leucopenia nor anemia in peripheral blood was observed, suggesting the decreased platelet count might have been due mainly to hypophosphatemia. Liver dysfunction leads to hypoproduction of coagulation cofactors[3], and the aggravation of our patient's coagulation disorder due to venous congestion resulted from extended bed rest caused DIC[4].

Phosphate is incorporated into phosphorylated intermediates required for protein synthesis, glycogen formation, and phospholipids in the cell membranes of newly forming cells. Hypophosphatemia is the main cause of RS and often leads to depletion of phosphorylated compounds (ATP, 2,3DPG, G-3PD), all of which are important for energy production and oxygen delivery, and may result in cardiac, neuromuscular, hematological, and respiratory compromise. Severe hypophosphatemia caused by refeeding is not rare; in fact, it occurs in 0.8% of all hospitalized adult patients[5]. Thompson et al. reported that an ordinary clinical symptom of hypophosphatemia has been reported as less than 1.0 mg/dl, and that hypophosphatemia occurred in 42% of medical and surgical patients given total parenteral nutrition containing phosphate[6]. Our patient's serum phosphate concentration appeared normal before the crisis, but her liver dysfunction rapidly became significant due to treatment. At that time, her serum phosphate concentration was 2.1 mg/dl, which was low compared to the normal level, but was not such severe hypophosphatemia as to present the clinical manifestations described above. Brooks and Melnik reported that a rapid decrease in the serum phosphate concentration is more important than the absolute serum concentration. They recommended that glucose administration should be restricted to 4–7 mg/kg/min and advised a supplementation rate of 31–62 mg (1–2 mmol)/100 kcal phosphate[7].

Upon admission, she was scarcely able to take meals. However, based on a counseling program and enteral nutrition, her caloric intake gradually increased, from 600 kcal/day at first to 1,000 kcal/day. We also prescribed parental nutrition beginning at 120 kcal/day, which increased to 300 kcal/day. Temporarily, her platelet decreased, but improved through treatment. After that, her body weight satisfactorily increased until hypoalbuminemia and heart failure on UCG, that is, RS became apparent. In one study, the need for hospitalization with close medical monitoring and slow oral refeeding was reported. Refeeding in hospitalized patients with AN has shown that a minimum 4-day weight gain criterion between 0.36 and 0.55 kg could be achieved without the accompanied complications of refeeding. Increased clinical disease severity (as measured by BMI) correlated significantly with increased bradycardia and decreased left ventricular forces. Therefore, we should have monitored her cardiac status daily since the first week of hospitalization[8]. Part of her weight gain might be due to excessive water intake. The dramatic change in body fluid might have contributed to the aggravation and more care should have been taken with water intake in addition to careful monitoring of her body weight gain. In short, fluid intake should be decreased as enteral nutrition is increased.

In anorexic patients, malnutrition causes fatty liver because of lipid metabolic disorder[9], but severe liver failure, as in our case, is rare. To treat RS, we prescribed 2.5 g of albumin and diuretics, but RS was aggravated as her heart condition worsened due to the refilling of extra vascular fluid into the dysfunctional ventricle which subsequently triggered acute liver dysfunction (AST 1914 IU/l, ALT 1582 IU/l), showing that in such a vulnerable condition, even though careful attention is paid to the reinstitution of nutrition to prevent development of RS, fluid retention is likely to occur, accompanied by the manifestation of dysfunction of various organ systems such as pericardial effusion and liver dysfunction. The pathology of the severe liver failure has been proposed. First, Carmela et al.[10]reported that in addition to the already existing heart condition, dehydration, hypotension, and bradycardia appeared, causing a microcirculation disorder of the liver. So the first step of therapies should be plasma volume support and monitoring of cardiac function. Then, congestion or ischemia of the intestinal mucosa due to the heart condition caused a break in the mucosal barrier, allowing endotoxins to enter the intestine, resulting in severe liver failure[11]. We regret that our therapeutic interventions such as albumin were too late and the diuretics might have been the trigger of the severe liver failure.

Brooks and Melnik proposed that RS is more than hypophosphatemia[7]. Complications secondary to refeeding are the result of perturbations in various electrolytes, vitamin deficiencies, glucose and fluid intolerance, and cardiac dysfunction. In cases such as ours where severe AN and RS are overlapping in part, care must be taken to make a distinction not to become confused. More careful consideration as to the possible onset of RS must be accompanied by more care in the control of rapid body weight gain. We propose that medical treatment should be initiated slowly to prevent dramatic changes in the patient's fluid and electrolyte status.
